# A Quantum Genetic Algorithm for Building a Semantic Textual Similarity Estimation Framework for Plagiarism Detection Applications

**DOI:** 10.3390/e25091271

**Published:** 2023-08-29

**Authors:** Saad M. Darwish, Ibrahim Abdullah Mhaimeed, Adel A. Elzoghabi

**Affiliations:** 1 Department of Information Technology, Institute of Graduate Studies and Research, Alexandria University, 163 Horreya Avenue, El Shatby, P.O. Box 832, Alexandria 21526, Egypt; adel.elzoghby@alexu.edu.eg; 2 Department of Computer Science, University of Technology, Baghdad 10082, Iraq; ibrahim.hjwel99@gmail.com

**Keywords:** plagiarism detection, semantic analysis, optimization, quantum evolutionary algorithms

## Abstract

The majority of the recent research on text similarity has been focused on machine learning strategies to combat the problem in the educational environment. When the originality of an idea is copied, it increases the difficulty of using a plagiarism detection system in practice, and the system fails. In cases like active-to-passive conversion, phrase structure changes, synonym substitution, and sentence reordering, the present approaches may not be adequate for plagiarism detection. In this article, semantic extraction and the quantum genetic algorithm (QGA) are integrated in a unified framework to identify idea plagiarism with the aim of enhancing the performance of existing methods in terms of detection accuracy and computational time. Semantic similarity measures, which use the WordNet database to extract semantic information, are used to capture a document’s idea. In addition, the QGA is adapted to identify the interconnected, cohesive sentences that effectively convey the source document’s main idea. QGAs are formulated using the quantum computing paradigm based on qubits and the superposition of states. By using the qubit chromosome as a representation rather than the more traditional binary, numeric, or symbolic representations, the QGA is able to express a linear superposition of solutions with the aim of increasing gene diversity. Due to its fast convergence and strong global search capacity, the QGA is well suited for a parallel structure. The proposed model has been assessed using a PAN 13-14 dataset, and the result indicates the model’s ability to achieve significant detection improvement over some of the compared models. The recommended PD model achieves an approximately 20%, 15%, and 10% increase for TPR, PPV, and F-Score compared to GA and hierarchical GA (HGA)-based PD methods, respectively. Furthermore, the accuracy rate rises by approximately 10–15% for each increase in the number of samples in the dataset.

## 1. Introduction

Over the last few decades, forensic linguistics has developed and used a type of language analysis that has helped put in place reliable ways to find plagiarism. Forensic linguistics research, which looks at how language affects the law, has shown that it is possible to figure out how likely it is that two or more texts were written independently. So, this analysis can be used as both a way to find out more and as proof, not just in legal situations but also in ethical ones [1,2,3,4,5]. Today, more and more cases of plagiarism are being reported. This could be because of one or more of the following: easy access to information; intense pressure to publish in academia for career advancement; lack of confidence and writing skills; or writing manuscripts quickly or under stress to meet a deadline. Also, because authors do not know what plagiarism is, they do not know that it is wrong to copy and paste word-for-word, even if they give a reference to the original text. Plagiarism detection (PD) methods look for text that is similar or the same between two or more documents [6]. As most plagiarists reuse the text from other source papers to disguise plagiarism by changing terms with synonyms or paraphrasing, and maybe by rearranging the sentences, detecting plagiarism can be a very difficult process. On the other hand, it has inspired the creation of automated detection methods. Publishing houses have recently shown an eagerness to combat plagiarism [7].

Current PD approaches might have some shortcomings that reduce their effectiveness in detecting plagiarized texts. Here are the issues [8]: (1) Most algorithms can only identify word-for-word plagiarism, while others can detect random alterations. Online PDs fail or lose efficiency at greater degrees of complexity [9]. (2) Plagiarists have it easier with automatic translators, summarizers, and other tools. (3) Idea plagiarism detection tools are ineffective [10]. (4) Most PD methods may not detect structural alterations [11]. (5) Passage-level detections may lack linguistic, semantic, and soft computing tools. Syntactic, semantic, structural, and linguistic features must be evaluated to reveal hidden obfuscations. (6) Finally, there are not enough benchmark data to evaluate plagiarism techniques [12]. Plagiarism can take place in two ways: (1) Literal plagiarism, in which the plagiarist uses all or part of another person’s work in their own. (2) Semantic plagiarism (intelligent) is when someone steals the content of another person’s work but uses different words to describe it.

Plagiarism can be as simple as copying and pasting or as complicated as changing the words around. See [8] for more information. Textual documents can be divided into two basic types based on how similar their languages are or how different they are. These are monolingual and cross-lingual (CL) [13,14]. There are not many ways to find CL plagiarism because it is hard to find closeness between two text segments in different languages [14]. Unlike its multilingual counterpart, monolingual plagiarism detection focuses on pairs of languages that are mutually exclusive, such as English and English. This kind of detection approach constitutes the vast majority [14]. Detection may be further subdivided into the intrinsic type and the extrinsic type based on whether or not external references are used. Intrinsic detection is a document analysis technique that identifies potentially harmful files based only on linguistic features such as authorial style, paragraph structure, and section formulations [8]. In extrinsic detection, the suspect document is compared to a database or collection of source documents.

Optimization is an interesting area of research. In general, there are two types of optimization solution methods: deterministic and stochastic methods. Every method has its own pros and cons [15]. In deterministic methods, the initial values of the parameters and the conditions completely determine the model’s output. Some randomness is built into stochastic methods [16]. Although various random approaches have been developed, such as swarm intelligence, genetic algorithms are becoming more popular for solving complex, large-scale optimization issues [17]. The quantum genetic algorithm (QGA) is an innovative evolutionary algorithm that combines quantum computing with conventional genetic algorithm technology. The approach can solve the same types of problems as the traditional genetic algorithm, but it does it far more quickly because of quantum parallelization and the entanglement of the quantum state, which speeds up the evolutionary process. A global search for a solution may be performed with quick convergence and a small population size by combining the probabilistic mechanism of quantum computing with the evolutionary algorithm. These methods have proven effective in a broad range of combinatorial and functional optimization problems [18,19,20].

### 1.1. Problem Statement

Even if that is true, putting plagiarism in a legal context is hard because you have to find strong proof that a suspicious text has been copied. When the text is copied and pasted word-for-word, it is usually enough to compare the suspect text to the possible source text to find the overlap. Most cases, though, are much more complicated. New ways to find plagiarism lead to new ways to avoid being caught, which in turn require new ways to find plagiarism. Plagiarism is when someone passes off someone else’s work as their own without giving credit. Plagiarism covers a wide range of things, from copying someone else’s words to copying someone else’s ideas. Recently, there have been many PD approaches based on semantic similarity and sentence-based concept extraction that may facilitate the discovery of paraphrases. To detect instances of plagiarism, several algorithms delve into the document’s semantic concept by analyzing factors like the author’s writing style, the structure of the paragraphs, the arrangement of the sections, etc. Obfuscated plagiarism cannot be prevented using these techniques, however.

### 1.2. Contribution and Methodology

In this paper, a modified PD algorithm is utilized to detect plagiarism using the semantic concept and the QGA. Adopting the QGA inside the PD model can facilitate the optimization of a similarity search. Furthermore, the QGA is employed to find sentences that briefly show the concept of the source document. On the other hand, semantic-level concepts are captured by applying semantic similarity metrics, which depend on the WordNet database for extracting semantic information. How successfully individuals are mapped to fitness metrics is what gives the QGA its usefulness in our context. Since all quantum individuals are reduced to a single solution during the measurement of the fitness function, the benefits disappear if the mapping is one-to-one. More individual-to-fitness mappings mean a higher potential diversity benefit for the QGA.

The remainder of this paper consists of the following sections: Some background on quantum genetic algorithms is briefly discussed in Section 2. The third section provides a literature review of relevant publications for the PD framework. The suggested approach is presented in Section 4. The assessment of the suggested technique, including results and discussion, is presented in Section 5. The study is concluded, and possible future directions are discussed in Section 6.

## 2. Preliminaries

In this section, we will go through the fundamental concepts of quantum genetic algorithms that will be used in the proposed framework. Primarily, evolutionary algorithms (EAs) are stochastic searches and optimization techniques inspired by the concepts of natural biological evolution. EAs have many advantages over more conventional optimization techniques, including their scalability, versatility, and independence from domain-specific heuristics. However, it is challenging to incorporate the characteristics of population diversity and selection pressure concurrently into EAs like the genetic algorithm (GA). In the face of rising selection pressure, the search narrows in on the best individuals in the population, but the resulting exploitation reduces genetic variety. The reason for this is that deterministic values are used in the definition of representations of EAs [20,21]. 

QGAs are a hybrid of conventional GAs and quantum algorithms. The superposition of quantum mechanical states, or “qubits”, is the primary foundation for these. Here, instead of being represented as a binary string, for example, chromosomes are vectors of qubits (quantum registers). This means that a chromosome may stand in for a superposition of all possible states. The QGA is distinguished by its simultaneous capacity for quick convergence and global search. Quantum computing concepts and principles like qubits and a linear superposition of states form the basis of the QGA [22,23]. One way to express the status of a qubit is as follows:

(1) |Ψ〉=α|0〉+β|1〉

(2) $${\left|\alpha \right|}^{2}+{\left|\beta \right|}^{2}=1$$

The probabilities of the qubit being in the ‘0’ and ‘1’ states are specified by the expressions ${\left|\alpha \right|}^{2}$ and ${\left|\beta \right|}^{2}$, respectively, where α and β are complex numbers describing the probability amplitudes of the two states. Information on the states of a system may be stored in a system of *m*-qubits. However, a quantum state collapses to a classical one upon observation [24]. For *m*-qubits, the representation is:

(3) α1β1α2β2….....αmβm,|αi|2+|βi|2=1,i=1,2,….,m

Consider a three-qubits system with three pairs of amplitudes:

(4) 121212−121232

The current system status may be represented by:

(5) $$\frac{1}{4}|000\rangle +\frac{\sqrt{3}}{4}|001\rangle -\frac{1}{4}|010\rangle -\frac{\sqrt{3}}{4}|011\rangle +\frac{1}{4}|100\rangle +\frac{\sqrt{3}}{4}|101\rangle -\frac{1}{4}|110\rangle -\frac{\sqrt{3}}{4}|111\rangle$$

This allows for eight possible states of information storage inside the three-qubit machine. Evolutionary computing with a qubit representation offers a more diverse feature than conventional approaches since it may express the superposition of states. While in classical representation at least eight chromosomes are needed to represent a state, just one qubit chromosome is needed to represent eight states. Convergence may also be attained using the qubit format. The qubit chromosome converges to a single state and loses its distinctive feature of diversity when either ${\left|\alpha_{i}\right|}^{2}$ or ${\left|\beta_{i}\right|}^{2}$ approaches 1 or 0. Therefore, it is possible for the qubit representation to have both exploratory and exploitation properties [24]. The structure of the QGA is described in Algorithm 1 [21,24].


**Algorithm 1: QGA Procedure**

Begin
 t=0 Initialize Q(t) Make P(t) by observing Q(t) states Evaluate P(t) Save the best solution among P(t) While (not termination-condition) do Begin   t=t+1   Make P(t) by observing Q(t−1) states   Evaluate P(t)   Update Q(t) using quantum gates U(t)   Store the best solution among P(t)
 End
End

The QGA maintains a population of qubit chromosomes, $Q\left(t\right)=\left\{q_{1}^{t},q_{2}^{t},q_{3}^{t},\ldots .,q_{n}^{t}\right\}$ at generation *t*, where *n* is the population size, *m* denotes the total number of qubits and indicates the string length of the qubit chromosome, and $q_{j}^{t}$ is the definition of a qubit chromosome:

(6) qjt=[α1tβ1tα2tβ2t........αmtβmt],j=1,2,…..,n

(7) $$|\Psi_{q_{j}^{o}}\rangle =\sum_{k=1}^{2^{m}}\frac{1}{\sqrt{2^{m}}}|S_{k}\rangle$$

(8) $$U\left(\theta \right)=\left[\begin{array}{cc} \cos\theta & -\sin\theta \\ \sin\theta & \cos\theta \end{array}\right]$$

where $S_{k}$ is the *k*-th state represented by the binary string $\left(x_{1}x_{2}\ldots \ldots x_{n}\right)$, $x_{i},\;i=1,2,\ldots .m$, is either 0 or 1, and θ is the rotation angle. The effectiveness (fitness) of each solution is ranked. Then, among the available binary options, the P(t) is chosen as the best possible starting point and saved. Q(t) uses the binary solutions and the best-stored solution to construct an updated solution, which is then processed via the relevant quantum gates U(t). To solve real-world issues, we may tailor the design of quantum gates to meet specific needs.

## 3. The State of the Art

Plagiarism often falls into one of three categories: (1) If the original texts are available, the study centers on comparing the suspect text(s) to the potential originals to uncover linguistic evidence to infer that the suspect text is truly a derivative or original; (2) if the source texts are unknown but plagiarism is suspected, the analysis focuses on determining whether the material in question is plagiarized or not based on its inherent stylistic evidence; or (3) if two or more texts are suspected of joint rather than individual composition, the linguistic study will center on determining whether any probable overlap between the texts is coincidental or the consequence of collaboration. Therefore, linguistic studies seek to determine whether instances of textual overlap across various papers are suggestive of plagiarism and if such overlap constitutes fraudulent behavior [1,2,3,4,5].

**Figure 1:**
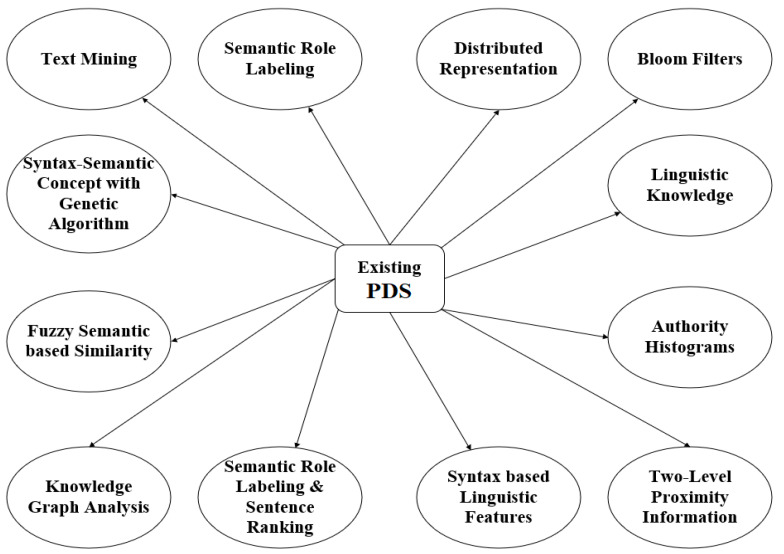
Taxonomy of existing PD models.

To aid in the building of the suggested model, this section discusses a few related PD models and plagiarism prevention efforts from the cited literature. Figure 1 shows the taxonomy of the existing PD models. In Ref. [25], the authors developed an approach based on Semantic Role Labeling (SRL) to determine semantic similarity between texts. All of WordNet’s ideas were combined into one node called the “topic signature node,” which instantly captures suspicious elements from documents. This method identifies copy–paste and semantic plagiarism, synonym substitution, phrase restructuring, and passive-to-active voice changes. Hence, since not all arguments impact the PD process, the fuzzy inference system should be used to increase the similarity score that argument weighting improves.

In Ref. [7], the authors studied sentence ranking for PD and SRL. Vectorizing the material generates suspicious and original sentence pairings. Pre-processing, candidate retrieval, sentence rating, SRL, and similarity detection are the five stages of the approach. The proposed technique leverages SRL to determine the semantic functions of each sentence word based on its verb. This depends on the word’s semantic meaning. The algorithm recognizes copy–paste, close copy, synonym substitution, phrase reordering, and active/passive voice conversion faster and more accurately. It is unknown what degree of syntax is required to provide a thorough study of semantic roles and how the state of the art constrains SRL tagging and parsing performance.

In Ref. [26], the semantic and syntactic relationships between words are integrated. This strategy improves PD because it avoids picking source text sentences with high similarity to suspect text sentences with dissimilar meanings. It can identify copied text, paraphrases, sentence translations, and word structure changes. This approach cannot discriminate between active and passive sentences, however. In Ref. [27], the authors suggested a fuzzy semantic-based similarity approach for detecting obfuscated plagiarism. After feature extraction, the text characteristics are entered into a fuzzy inference system, which models semantic similarity as a membership function. Once the rules have been evaluated, the results are averaged to obtain a single score that indicates how similar two texts are. The technology detected literal and disguised plagiarism. The system cannot generalize and is not resilient to topological changes. Such modifications need rule-based adjustments and an expert to develop inference rules.

Another approach was suggested in [28] which treated document-level-text PD as a binary classification issue. The original source of a document was identified and that information was used to determine whether or not the document in question contained plagiarized content. The main parts are feature extraction, feature selection, and classification using machine learning. After pre-processing and filtering, part-of-speech (POS) tags and chunks removed extraneous data. The method investigated the influence of plagiarism categories and complexity on attributes and behavioral variances. The lack of a large database of manual plagiarism instances is a concern; thus, creating one is necessary for testing detection techniques.

The work in [8] presented another effort to identify plagiarism. The described study explores GA syntax–semantics concept extractions to detect idea plagiarism. Pre-processing, GA source sentence extraction, document level, and passage level are the four major components. Natural language processing (NLP) approaches are utilized for word-level extraction within documents. Sentence-based comparisons employing integrated semantic similarity metrics are employed in the passage-level identification step. Using passage boundary conditions, the passage level is detected. In the offered technique, the concept of plagiarism enforced via summarizations is emphasized. The results demonstrated substantial performance in catching plagiarized texts. Plagiarism may also occur via elaboration and paraphrase, etc., which the system cannot detect.

In order to find instances of plagiarism, the study in [29] constructed a cutting-edge system that relies on semantic properties. For each possible suspect and source phrase combination, the system generates a relation matrix that uses semantic characteristics to calculate the level of similarity. This study presents two weighted inverse distance and gloss Dice algorithms that illustrate different text qualities (e.g., synonyms) and develops a novel similarity metric for plagiarism detection, which overcomes the limits of the current features. In addition, this study examines the efficacy of individual characteristics in identifying copied works, combining the most effective ones by giving varying weights to their individual contributions to further improve the system’s performance. The inverse weighted distance functions have a drawback in that the function must have a maximum or minimum at the data points (or on a boundary of the study region).

The study given in [30] outlines a three-stage process that, together, provides a hybrid model for intelligent plagiarism detection: initially, we cluster the data; then, we create vectors inside each cluster according to semantic roles, normalize the data, and compute a similarity index; and lastly, we use an encoder–decoder to provide a summary. For the purpose of choosing the words to be used in the production of vectors, K-means clustering, which is calculated using the synonym set, has been proposed as a method. Only if the last stage’s estimated value is greater than a threshold value is the following semantic argument evaluated. A brief description is generated for plagiarized documents if their similarity score is high enough. The experimental results demonstrated the effectiveness of the strategy in identifying not only literal but also connotative and concealing forms of concept copying. However, long sequences take a long time to process because of the slowness of the neural network’s processing and the difficulty of training it if activation functions are used. Finally, it has problems like gradient vanishing and explosions.

In Ref. [31], the authors introduced an efficient method for determining the structural and semantic similarity between two publications by only analyzing a subset of the material of each document instead of the whole thing. To improve plagiarism detection regardless of word order changes, a collection of remarkable keywords and different combinations are used to compute similarity. The importance of a word varies depending on where in the article it appears. As a final step, a weighted similarity is determined using an AHP (Analytical Hierarchy Process) model. It was shown that the suggested method outperformed its competitors in terms of runtime and accuracy for detecting semantic academic plagiarism. One potential drawback of the AHP is the high number of pairwise comparisons it requires. This is due to the fact that comparing each criterion and then each option with regard to a given criterion is required.

In Ref. [32], the authors offered an approach to detecting two common forms of paraphrased text: those that involve the use of synonyms and those that use the reordering of words in plagiarized sentence pairs. They introduced a three-stage technique that makes use of context matching and pertained word embedding to detect instances of synonymous replacement and word reordering. Their experiments revealed that the Smith–Waterman method for plagiarism detection combined with ConceptNet batch-pertained word embedding yields the highest scores. Methods to determine paraphrase styles for plagiarism detection may be used from this study to supplement similarity reports from existing plagiarism detection systems. Even though it is the most sensitive technique for detecting sequence similarity, the Smith–Waterman approach does not come without its price. Time is a major restriction, as conducting a Smith–Waterman search requires a lot of processing power and time.

Two methods for identifying external plagiarism are provided in [33]. Both methods use a bag-of-words strategy-based two-stage filtering procedure, first at the document level and then at the sentence level, to reduce the search area; only the outputs of both filters are then evaluated for plagiarism. One uses the WordNet ontology and the term frequency–inverse document frequency (TF-IDF) weighting technique to create two structural and semantic matrices; the other uses a pre-trained network technique of words embedding fast text and TF-IDF weighting to create the same outcome. After forming the aforementioned matrices, the structural similarity of the weighted composition and the Dice similarity are used to determine the degree of similarity between the pairs of matrices representing each phrase. The similarity between the suspect text and the minimum criterion is used to classify documents as plagiarism or non-plagiarism. Using the PAN-PC-11 database, the authors conducted experiments to determine whether or not a word embedding network, as opposed to the WordNet ontology, would be more successful in detecting instances of extrinsic plagiarism. However, TF-IDF weighting does have certain restrictions. It may be time-consuming for large vocabularies since it calculates document similarity directly in the word-count space. It assumes that evidence for similarity may be found in the counts of various terms. One potential problem with the adaptable layout described above is that WordNet’s’ meaning and scope might quickly diverge from one another. We cannot be sure that we will be encoding the same relationships or that we will be covering the same conceptual ground [34,35].

In Ref. [36], the authors created a new database that contains all the characteristics that indicate various linguistic similarities. As a solution to textual plagiarism issues, the developed database is offered for use in intelligent learning. The produced database is then used to propose a deep-learning-based plagiarism detection system. During development, many deep learning techniques, including convolutional and recurrent neural network topologies, were taken into account. To assess the efficacy of the presented intelligent system, comparison research was conducted using the PAN 2013 and PAN 2014 benchmark datasets. In comparison to state-of-the-art ranking systems, the test findings demonstrated that the suggested system based on long short-term memory (LSTM) ranked first. However, LSTMs are easy to overfit and are sensitive to different random weight initializations.

Using the fuzzy MCDM (multi-criteria decision-making) technique, the research in [37] compared and contrasted many academic plagiarism detection strategies and offered guidelines for creating effective plagiarism detection tools. They described a framework for ranking evaluations and analyzed the cutting-edge methods for detecting plagiarism that may be able to overcome the limitations of the state-of-the-art software currently available. In this way, the research might be seen as a “blueprint” for developing improved plagiarism detection systems. An innovative and cutting-edge technique known as compressive sensing-based Rabin Karp is offered for use in the system presented in [38]. This technique calculates both syntactic and semantic similarities between documents using a sampling module to shrink the dataset and a cost function to identify document repetition. Yet, simply applying the hash function based on the generated table may result in cases where the hash codes for the pattern and text are the same, yet the pattern’s characters do not match those in the text. For current surveys that include the most up-to-date research in the plagiarism detection area, please refer to [39,40].

A novel plagiarism detection approach is presented in [41] to extract the most useful sentence similarity features and build a hyperplane equation of the chosen features to accurately identify similarity scenarios. The first phase, which contains three steps, is used to pre-process the papers. The second phase is dependent on two different strategies: the first strategy relies on the standard paragraph-level comparison, while the second strategy relies on the calculated hyperplane equation utilizing Support Vector Machine (SVM) and Chi-square methods. The best plagiarized segment is taken out in the third step. On the whole test corpus of the PAN 2013 and PAN 2014 datasets, the recommended approach attained the best values of 89.12% and 92.91% of the Plagdet scores and 89.34% and 92.95% of the F-measure scores, respectively.

The present plagiarism detection solutions now on the market compare plagiarism only when the input document includes text, despite the fact that there are a number of tools available that address the issue of plagiarism using various methodologies and features. However, when the input document is an image, the techniques currently in use do not check for plagiarism. The authors in [42] suggested a tool that searches both the text and text hidden in images using an exhaustive searching approach. The project’s suggested tool compares the input document’s content to that of websites and returns findings on how similar they are. The source and suspect papers are in two different languages, making it difficult to identify cross-lingual plagiarism (CLP). In this context, a number of solutions to the issue of CPD in text documents were proposed. To obtain comparability metrics, the authors in [43] employed the one-gram and tri-gram of the pre-processed text. The models are constructed using five ML classifiers: KNN, Naive Bayes, SVM, Decision Tree, and Random Forest. The trial demonstrates that KNN, RF, and other models offer superior outcomes versus other models.

Commercial plagiarism detection tools are accessible online for purchase or subscription. EVE2, Plag Aware, Write Check, Turnitin, and Ithenticate are some of the most well known [44]. Turnitin is an online similarity detection service that compares submitted papers to various databases using a proprietary algorithm to check for possibly plagiarized material. In addition to scanning its own databases, it has licensing arrangements with significant academic private databases. Turnitin does not deal with the causes of academic integrity problems, and so it does not fix them. Instead, it might give students the impression that they are being held accountable for cheating from the very first day of class or that their work is being used against them and others without their permission. iThenticate is a plagiarism prevention tool that assesses written material (such as journal article manuscripts, proposals, research reports, theses, and dissertations, among other things) against millions of published works that are accessible online and via paid databases. The following are some benefits of iThenticate: The finest tool for detecting plagiarism in academic writing is iThenticate, which employs cutting-edge algorithms to evaluate submitted text against a huge library of scholarly publications.

Despite decades of study, PD might be strengthened to better prevent intellectual property theft. Still, PD should account for things like running time and computational complexity. The available PD approaches are not all suitable to be employed in all applications. To address these issues and outperform competing methods, a model combining semantic idea extraction and the QGA for optimizing similarity search has been proposed. The QGA is structurally similar to classical genetic algorithms, with the exception that quantum gates and quantum superposition are used to construct the initial and updated populations, with consideration given to the adaptation of such operators to meet GA-based PD issues. One clear benefit of a QGA is that its population tends to be more diverse than that of a non-QGA. To put it another way, a quantum population may be exponentially greater than its “size” in the classical world. Only one possible solution may be represented by each individual in a classical population. Each “individual” in a quantum population is a superposition of many different possible solutions. In this sense, the population of a quantum system is far greater than that of a classical system.

## 4. The Proposed QGA-Based Plagiarism Detection Model

**Figure 2:**
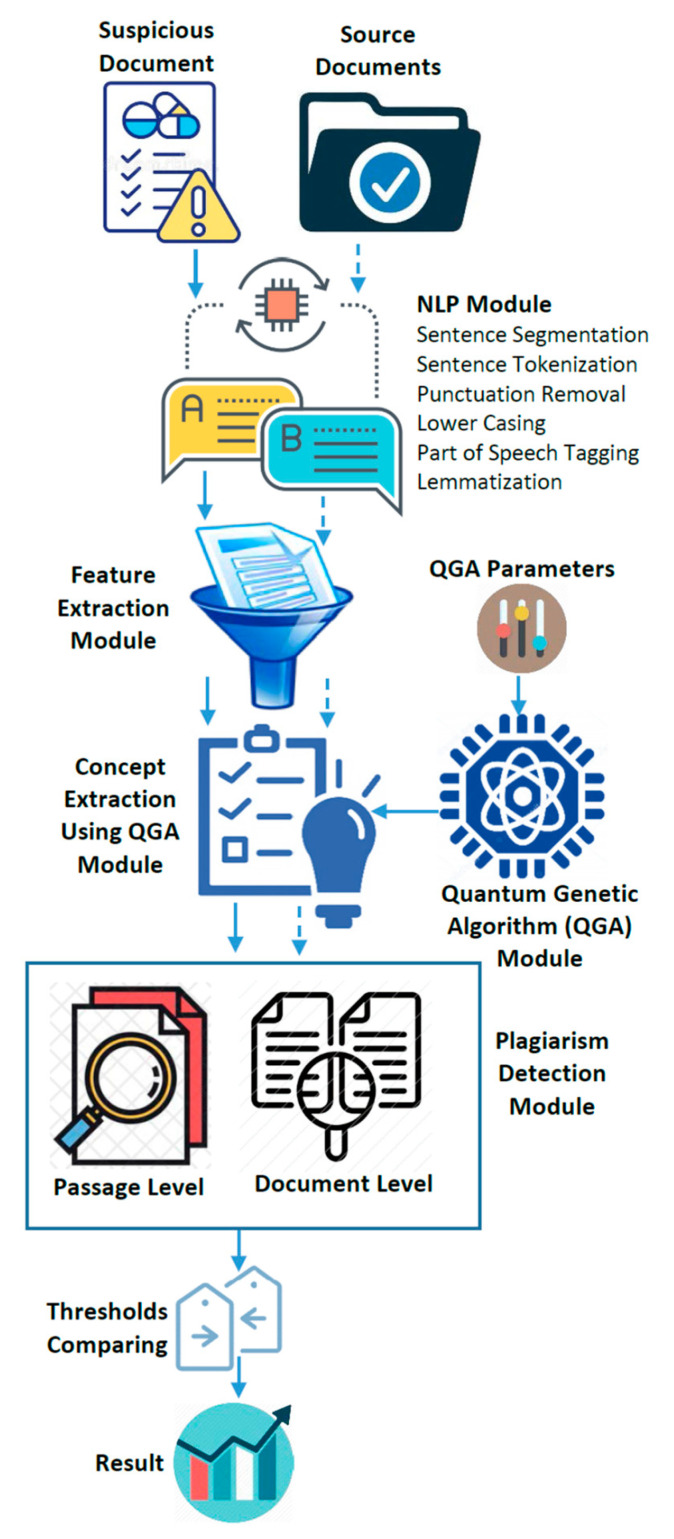
The proposed plagiarism detection framework using the QGA.

This section presents the suggested model for QGA-based idea (semantic) extraction for plagiarism detection. PD exploits document notions at several structural levels for document-level (DL) and passage-level (PL) detection. QGA-based sentence scoring is examined for sentence-level extraction. The DLD stage captures nouns and verbs using natural language processing (NLP) methods. In the PLD phase, phrase-based assessments utilizing a joint similarity measure with WorldNet detect plagiarized sentence pairings. We decided to use a quantum-inspired evolutionary algorithm to solve the PD problem because of the many benefits of quantum-inspired metaheuristics. (1) With quantum gates and quantum parallelism, it is possible to compute all possible values of a given variable simultaneously, which not only enhances the quality of the result but also drastically shortens the search time. (2) The use of quantum superposition and quantum gates to represent individuals in a population results in (a) more diversity, (b) enhanced search capacity, (c) faster and more accurate convergence, and (d) efficient escape from local optima. Due to the limited number of individuals, the method may quickly and efficiently probe the search space for a global solution, even if it only contains a single element. (3) There is a balance between diversification/intensification and exploration/exploitation [18,21,22]. Figure 2 shows the suggested framework, and each module is discussed in the following subsections.

### 4.1. Pre-Processing and Document Representation 

The database, which includes both source and suspect documents, is pre-processed in the first module. The steps included in this section are as follows.

#### 4.1.1. Sentence Segmentation and Tokenization 

First, suspicious ${(X}_{susp})$ and source ${(X}_{src})$ documents are sentence-segmented. Text segmentation is a pre-processing procedure that divides text into meaningful components like sentences or words. The document is split into sentences. Then, source and suspect phrases are tokenized. Punctuation and capitalization are eliminated [7,8,27].

#### 4.1.2. Part-of-Speech Tagging and Lemmatization

After the pre-processing step, tokenized words are employed for the part-of-speech (POS) tagging of suspect and source tokenized phrases. Each word is labelled as a noun, verb, adjective, preposition, etc. Noun, verb, adjective, and adverb tags are the only semantic tags that are kept. Conjunctions, prepositions, articles, pronouns, and determinants were taken out of the sentence, along with anything else that did not add meaning. By conserving memory and speeding up processing, removing such words improves accuracy and time. Lemmatization reduces words to their dictionary base forms and allows for comparisons. The Stanford Log-Linear Speech Tagger and WordNet Lemmatizer were employed for POS tagging [8,45]. The pre-processed suspicious sentence in $X_{susp}$ is $S_{susp}$, while the source sentence in $X_{src}$ is $S_{src}$. Each pre-processed source and suspect tokenized sentence includes lemmatized and POS-tagged words available for feature extraction [7,8,27,45].

#### 4.1.3. Feature Extraction

The pre-processed source and suspect documents are a collection of tokenized sentences, and the Vector Space Model (VSM) with term-frequency-inverse sentence frequency (tf−isf) weighting reflects the vocabulary of the lemmatized and POS-tagged words contained in these documents [8]. (tf−isf) is a metric developed for use in information retrieval (IR) that attempts to quantify a word’s significance within the context of a phrase [28,45,46,47,48]. The w(t,S) weight is calculated using:

(9) tf(t,S)=f(t,S)

(10) $$isf(t,X)=log\frac{\left|X\right|}{\left|\{S\in X;t\in S\}\right|}$$

(11) w(t,S)=tf(t,S)∗isf(t,X)

The number of times a term t appears in any generic sentence S is denoted by term frequency tf(t,S). The term-inverse sentence frequency (isf) is used to highlight the fact that the computation is performed over individual sentences as opposed to whole documents, where X is the collection of all sentences found in the provided documents. Sentence vectors for the source and suspect sentences are denoted by $\vec{s_{src}}$ and $\vec{s_{susp}}$, respectively. 

### 4.2. The Quantum Genetic Algorithm for Extracting Sentence Concepts

Concept extraction using the QGA is feasible when the documents have been pre-processed and expressed in tf−isf weight form. The documents’ syntactic concepts are derived from their respective structural levels. Paragraphs, phrases, sentences, and keywords are all ways in which these ideas may be found across a document [49]. The suggested approach starts by using sentence scoring methods with the QGA to extract sentence-based ideas from the original documents. In order to simplify the content of a lengthy text into a few carefully chosen sentences, the QGA is used.

#### 4.2.1. Population Initialization

Pre-processed source sentences, each of which will be given a fixed score, are the QGA’s input. Static scores, together with relevance and theme scores, may be calculated. Sentence weights are assigned to each $S_{src}$ in $X_{src}$ by extracting features from $X_{src}$ based on w(t,S). Both the relevance score and the thematic score may achieve this [47,48].

- Relevance Score

The relevance score expresses $S_{src}$ using if−isf weights, which is the source sentence’s pre-processed word count:

(12) $$Rel(S_{src})=\frac{\sum_{i=1}^{\left|S_{src}\right|}w\left(t_{i},S_{src}\right)}{\left|S_{src}\right|};Rel(S_{src})[0,1]$$

where $w(t,S_{src})$ denotes the sum of the tf−isf weights of each word t in $S_{src}$ and $\left|S_{src}\right|$ is the source sentence length.

- Thematic Score

The themeatic score is calculated by retrieving and sorting the words from the pre-processed $X_{src}$. The top L words are then saved in the $X_{src}$ keyword set $kw(X_{src})$:

$$Thm\left(S_{src}\right)=\frac{\left|kw\left(S_{src}\right)\right|}{L};$$

(13) $$Kw\left(S_{src}\right)=\{t|t\in S_{src}\wedge t\in kw(X_{src})\};Thm(S_{src})\in [0,1]$$

where $\left|kw(S_{src})\right|$ is the number of words between $kw\left(X_{src}\right)$ and $S_{src}$ in $X_{src}$ and $kw(X_{src})$ has L words. After calculating the relevance and thematic scores, $Stat\left(S_{src}\right)$ is calculated.

(14) $$Stat(S_{src})=Rel(S_{src})+Thm(S_{src});Stat(S_{src})\in [0,2]$$

**Figure 3:**
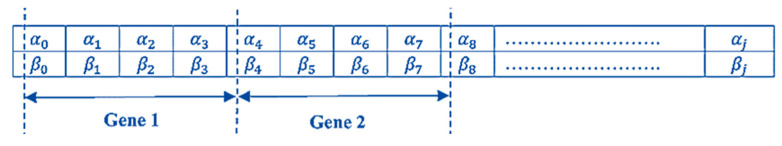
Quantum chromosome structure.

The $S_{src}$ with the associated $Stat(S_{src})$ will be employed for building the QGA population. A population with *N* chromosomes is randomly chosen. A chromosome is conceptually equivalent to a quantum register made up of a string of *m*-qubits. A quantum chromosome’s structure can be seen in Figure 3. All qubit amplitudes may be conveniently set to the value $1/\sqrt[{2}]{2}$ [22] to generate the starting population. This implies that each of the possible quantum superposition states is equally represented in a chromosome. To begin, we create *N* quantum registers and give them the labels ${Reg1}_{0}$ through ${Reg1}_{N-1}$, where *N* is the total number of individuals in the population. Then, each of these registers is layered on top of one another to create a superposition of all potential individuals. This means that each register is capable of storing all potential individuals. The next step is to apply the fitness function to each of the *N* quantum registers, and then store the results in a second set of *N* quantum registers, which are designated by the labels ${Reg2}_{0}$ through ${Reg2}_{N-1}$. The application of the fitness function will result in an entanglement being produced between the first set of registers and the second set of registers.

#### 4.2.2. Fitness Function Computation 

The quality of each quantum chromosome in the population is quantified at this stage in order to facilitate reproduction. A superposition of all the individuals who may have been there is included in each of the initial registers. Because of this, the data stored in each of the second registers is a superposition of all of the feasible fitnesses. Even if every individual was examined, which led to the generation of every fitness, there was still only one instance of the fitness function that needed to be applied to each register. The parallelism of quantum mechanics may be shown here [22,23,24]. The optimal solution would be to evaluate the highest fitness in register ${Reg2}_{i}$, which would then cause register ${Reg1}_{i}$ to collapse into a superposition of perfect individuals. The outcome of a measurement is completely unpredictable, and the probabilities are based on the amplitudes of the probabilities. Therefore, the likelihood of achieving a maximum level of fitness (Fit(C)) is precisely the same as the probability of accidentally producing an ideal individual. In our case, the fitness function (Fit(C)) is calculated as follows:

(15) $$Fit(C)=\sum_{i=1}^{\left|C\right|}Tot\left(S_{src}\right)$$

in which, a dynamic cohesiveness factor is generated for each phrase in C and supplemented using $Stat(S_{src})$. The cohesiveness factor determines sentence relatedness [50]. Cosine similarity measures lexical cohesiveness [51]. Cosine similarity between the source sentence vectors is calculated first.

(16) $$\mathrm{Cos}\left(\vec{S_{srci}},\vec{S_{Srcj}}\right)=\frac{\vec{S_{srci}}\cdot \vec{S_{Srcj}}}{\left||\vec{S_{srci}}|\right|\left||\vec{S_{Srcj}}|\right|};\forall i,j\vec{S_{srci}},\vec{S_{Srcj}}\in C$$

$\mathrm{Cos}\left(\vec{S_{srci}},\vec{S_{Srcj}}\right)$ denotes the cosine similarity between an $S_{src}$ vector pair $\left(\vec{S_{srci}},\vec{S_{Srcj}}\right)$ such that each sentence is a chromosomal *C* element. Cosine similarities are calculated and stored in a symmetric matrix with diagonal entry 1. The $S_{srci}$ sentence cohesion factor is then calculated.

(17) $$Coh\left(S_{srci}\right)=\frac{\sum_{j=1,j\neq i}^{\left|C\right|}\cos\left(S_{srci},S_{srcj}\right)}{\max\{\left(S_{srci},S_{srcj})\right\}},\forall_{ij}=\{1,2,...,|C|\},i\neq j$$

To avoid self-similarity, i≠j is used; otherwise, the denominator is 1. After computing the sentence cohesiveness factor, the total score for each source sentence $Tot\left(S_{src}\right)$ is determined.

(18) $$Tot(S_{src})=Stat(S_{src})+Coh(S_{src})$$

Using quantum selection and crossover, the fitness value *C* is used to build the next generation.

#### 4.2.3. Quantum Selection and Crossover 

Our initial population will be represented by a set of *N* paired registers, with half of the registers carrying fitness values and the other half having the superposition of individuals based on those fitness values. Normal procedures are followed upon crossover. The information included in the register ${Reg1}_{i}$ is combined with the information found in the register ${Reg1}_{j}$. Since both registers already contain a superposition of individuals, we obtain two additional superpositions as a result. In particular, if ${Reg1}_{i}$ contains all individuals with fitness values $Fit(C_{i})$ and ${Reg1}_{j}$ contains all individuals with fitness values $Fit(C_{j})$, then the superposition of all individuals that may be generated by crossing at the given location is achieved. The *N* registers (${Reg1}_{0}$ through ${Reg1}_{N-1}$) will then be subjected to the fitness function. The second set of registers is used to store the results and is entangled with the first set of registers in the same way that the initial population was. The next step is to take a measurement. This reduces the number of individuals from ${Reg1}_{0}$ through ${Reg1}_{N-1}$ to only those with the measured fitness, and it also collapses the superimposed fitness values to a single value. The generation ends when a selection is performed based on the calculated fitness values. Any desired mutations may be included [49,52,53].

**Figure 4:**
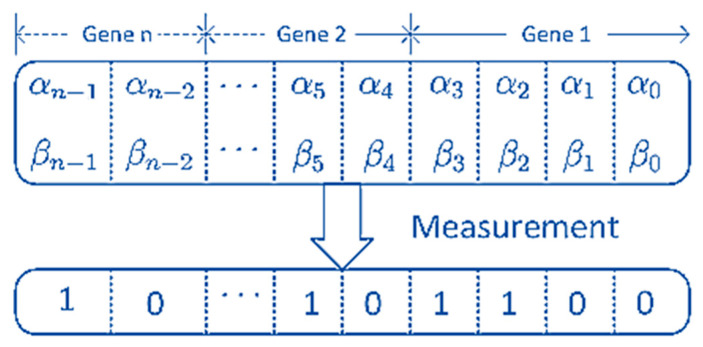
Measured chromosome.

Obtaining a result is the last action when the termination condition is met. The final product will be *N* pairs of registers, where each pair’s first register has a set of superimposed individuals with the same fitness value, and is entangled with the second register of the pair, which has the measured fitness value. A measurement of the first register will be able to identify one of the individuals as having the specified fitness level. This provides the effect that was sought, which is a single individual of the fitness level that was specified. It is necessary to conduct an observation on each qubit if we are to successfully utilize the superposed states of qubits (measuring chromosomes). Because of this, we are able to obtain a traditional chromosome, as illustrated in Figure 4. The purpose of this is to make it possible to evaluate each quantum chromosome. A final set of best C is generated, where the highest Fit(C) is picked, representing the best source sentence set $S_{src\_sel}$ [8].

**Figure 5:**
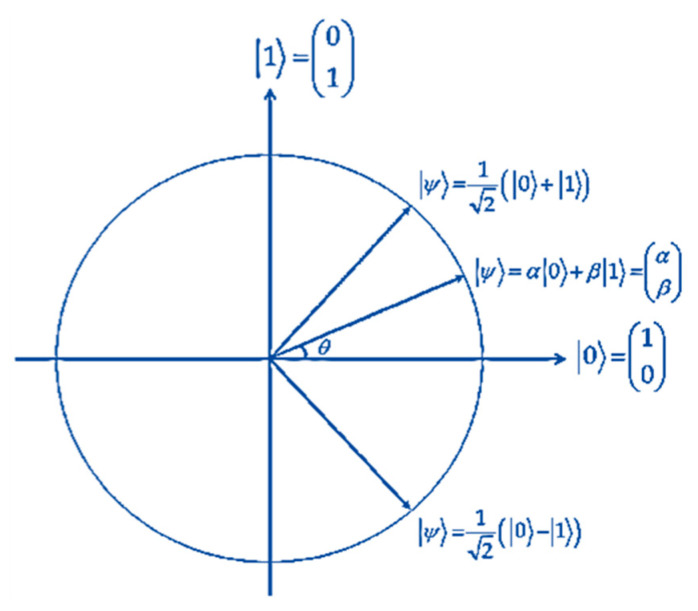
Qubit transformations with Hadamard gate.

The interference operation allows for the modification of specific amplitudes in order to optimize performance. It mostly entails shifting the state of each qubit in the direction of the optimal solution’s value. This is important for narrowing down the search for the best option. The amplitudes $\left(\alpha_{i},\beta_{i}\right)$ and the value of the corresponding bit in the reference solution determine the angle of the rotation that may be carried out using a unit transformation. Early convergence may be prevented by appropriately setting the rotation angle δθ. The direction of the change is determined by the values of $\alpha_{i}$, $\beta_{i}$, and the qubit inserted at location *i* in the individual (chromosome) being altered, all of which are typically estimated experimentally. The population Q(t) is revised when the qubits making up individuals are rotated using quantum gates. Equation [22] explains the rotation method that is employed:

(19) $$\left[\begin{array}{c} \alpha_{i}^{t+1}\\ \beta_{i}^{+1} \end{array}\right]=\left[\begin{array}{cc} \cos(\delta \theta_{i}) & -\sin(\delta \theta_{i})\\ \sin(\delta \theta_{i}) & \cos(\delta \theta_{i}) \end{array}\right]\left[\begin{array}{c} \alpha_{i}^{t}\\ \beta_{i}^{t} \end{array}\right]$$

where $\delta \theta_{i}$ is the rotation angle of each quantum chromosome’s qubit quantum gate *i,* as illustrated in Figure 5 [53]. As stated in [22], it is frequently taken via a lookup table to guarantee convergence; see Table 1.

The *i*-th bits of *x* and *b* (the optimal solution) are denoted by $x_{i}$ and $b_{i}$, respectively. The rotation angle $\theta_{i}$ has a sign that may be written as $S(a_{i}$,$b_{i})$, and *f* is the fitness function. Using the lookup table, we can see that this method increases the amplitudes of poor qubits according to angle $\delta \theta_{1}=0.08\pi$, while decreasing the amplitudes of good qubits according to angle $\delta \theta_{2}=0.001\pi$. Quantum bit amplitudes are adjusted in accordance with the signs of the amplitudes, the optimal solution, and the solution extracted with the respective container. Because reducing amplitudes only helps to correct stochastic mistakes, preventing genetic drift and guaranteeing genetic diversity, it stands to reason that $\delta \theta_{1}>\delta \theta_{2}$ [22].

### 4.3. The Document-Level Plagiarism Detection Phase

After selecting the important sentence-level ideas, the word-level concepts are retrieved. As most document ideas are transmitted using nouns and verbs, $S_{src\_sel}$ picks out nouns and verbs [8]. $S_{susp}$ collects nouns and verbs from each $X_{susp}$. The number of common source and suspect word ideas is utilized to detect document-level plagiarism in the DLD phase. If the count value is more than the threshold ε, the document is deemed to be plagiarized. After DLD, suspicious source document pairings that are determined as plagiarized proceed to the PLD phase.

### 4.4. The Passage-Level Plagiarism Detection Phase 

Semantic concept extractions are used for passage-level comparisons to calculate semantic similarity. Plagiarized suspicious source pre-processed document pairings are given to PLD. In this step, suspicious sentences are compared to $S_{src\_sel}$. The source sentences result from QGA’s sentence-level idea extraction. Since sentences are pre-processed, unnecessary words are deleted and each word is tagged (POS tag). For sentence comparisons, WordNet extracts semantic-based word synsets. Synsets are groups of semantically similar data elements [28]. POS information is compared to determine if a suspicious source sentence pair $\left(S_{susp},S_{src\_sel}\right)$ is plagiarized. That implies only comparing nouns and verbs, etc. Comparing word classes seems meaningless.

For each suspicious source word pair $\left(w_{q},w_{k}\right)$, WordNet is used to derive the synset lists $W_{q\_syn}$ of $w_{q}$ and $W_{k\_syn}$ of $w_{k}$ of each word. Only synsets in the same POS class as the word are retrieved for these lists. Common words between suspicious source sentence pairs $\left(S_{susp},S_{src\_sel}\right)$ are calculated and kept in the list *Count*. Suspicious word $w_{q}$ is checked for in $S_{src\_sel}$. The synonyms of $w_{q}$’s are taken from WordNet if it is not in $S_{src\_sel}$. $Syns(w_{q})$ represents a suspicious word’s synonym list. Common words between $Syns\left(w_{q}\right)$ and $S_{src\_sel}$ are calculated and added to *Count*, which includes the number of frequent terms or synonyms between suspicious and source sentence pairs. Using threshold τ, a suspicious source sentence combination is found to be plagiarized or not. If WorldNet’s similarity score is higher than the set value, the phrases are plagiarized [54]. Algorithm 2 outlines the main steps of the suggested technique.


**Algorithm 2: QGA for Plagiarism Detection**
*Input:* Dataset *X_src_*; Suspicious Document *X_susp;_* QGA Parameters, WordNet 
1-while *n* < size of documents do2-  *S* ← Sentence Segmentation (*X_src_*)3-  *y* ←04-  While *y* < *S* ! = NULL do5-  *T* ← Tokenization (*S*)6-  *z*←07-  while *z* < size of *T* do8-   *M* ← POS Tagging (*T*)9-   *N* ← Lemmatization (*M*)10-   *z*++11-  end12-  tf-isf (*N*)13-  *y*++14- end15- *n*++16-end17-*t* ← 018-while *termination condition not satisfied* do19-  *t* ← *t*+120-  Call Algorithm 1   // *QGA Procedure*21-  Return *Best_Pop* ←*New_Pop // Store the best solution among P(t)*22-end23-*sim*_1_← sum of words in *X_susp_ // the number of common word-level concepts in Xsusp**    that collects nouns and verbs*24-*sim*_2_← sum of words in *X_src_ // the number of common word-level concepts in Xsrc*25-

$\mathrm{If}\;{sim}_{1}-{sim}_{2}>\varepsilon$

26- Doc. Status = =Plagiarized27-end28-For each suspicious-source word pair (*w_q_*,*w_k_*) //*To compute the semantic similarity*29-   -$\;\mathrm{WordNet}\;\mathrm{is}\;\mathrm{used}\;\mathrm{to}\;\mathrm{derive}\;\mathrm{the}\;\mathrm{synset}\;\mathrm{lists}\;W_{q\_syn}\;\mathrm{of}w_{q}\;\mathrm{and}\;W_{k\_syn}\mathrm{of}\;w_{k}$30-   of each word.31-   - Only synsets in the same POS class as the word are retrieved for these lists32-end33-*Count* ←The common words between the compared suspicious-source sentence $\mathrm{pair}(S_{susp},S_{src\_sel})$ // $S_{src\_sel}$ *is the best set of selected source sentences**   extracted from QGA’ procedure*34-If count > *τ*35- Doc. Status = = Plagiarized36-end37-Else 38- Doc. Status = = not plagiarized39-end40-Output = Doc. Status


## 5. Experimental Results 

**Figure 6:**
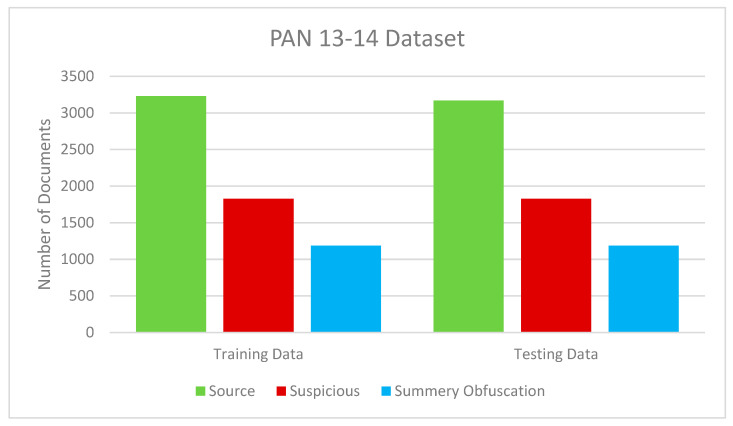
Size of training and testing datasets.

The effectiveness and reliability of the suggested model were evaluated using MATLAB implementation and QuTiP package Release 4.7.1 [55] for building quantum genetic algorithm modules. The prototype verification method was developed in a modular form and tested on a DellTM InspironTM N5110 Laptop, Dell computer Corporation, Texas, which included the following specifications: 64-bit Windows 7 Home Premium, 4.00 GB RAM, Intel(R) Core(TM) i5-2410M CPU, 2.30 GHz. Benchmark datasets [56] provided the source for these data. Table 2 displays the proportion of plagiarized to original papers in each group of suspects. The Summary Obfuscation (SO) training and test datasets provided by the PAN13-14 text alignment task were utilized to evaluate the plagiarism detection (PD) model in Figure 6. Different performance metrics, as shown in Table 3, were employed to assess the performance of the suggested model [57]. All test data examples may be predicted by a binary classifier as positive or negative. Table 4 displays the current QGA setup settings.

### 5.1. Experiment 1: A Comparative Study of the Different Types of GA

To validate the benefits of implementing the proposed model (semantic concept extractions with the QGA) for PD, this experiment compares the suggested model with related PD models that include syntax–semantic concept extractions with the GA [8] and syntax–semantic concept extractions with the hierarchical GA (HGA) [58]. The experiment was reported for datasets PAN13-14 in terms of TPR, PPV, and F-Score for all the used datasets. It is observable that the results of the QGA-based PD model are better than those that depend on both the HGA and the traditional GA. Table 5 reveals the superiority of the suggested model for document detection in terms of TPR, PPV, and F-Score. The recommended PD model achieves an approximately 20%, 15%, and 10% increase for TPR, PPV, and F-Score compared to the GA and HGA, respectively. These results might be explained by the fact that the proposed methodology uses semantic idea extraction to identify instances of plagiarism. Additionally, using the QGA aids in efficiently removing the non-plagiarized documents. It also lowers the number of PLD-phase sentence comparisons.

### 5.2. Experiment 2: QGA-Based PD Model Validation

The purpose of these tests was to verify the QGA’s usefulness in the features selection module by measuring its effect on accuracy. In this investigation, the adaptive feature selection technique is used to focus in on the most relevant details for enhancing the PD model. It compares the GA-based PD and the proposed QGA-based PD model for different datasets and provides a confusion matrix for all used datasets. The definitions regarding the confusion matrix are summarized in Table 6 [8]. Table 7 and Table 8 reveal that the QGA for PD achieves better results with the confusion matrix compared to the GA procedure. The QGA produces an approximate increase (of about 5%, on average) in plagiarism detection compared to the GA. The way the QGA works is that it facilitates the capturing of the non-plagiarized documents efficiently. Moreover, the QGA decreases sentence comparison numbers in the PLD. Utilizing the space’s desirable features is a discriminatory way to highlight individual differences. The feature selection issue is often multi-modal since there are often numerous optimal solutions. That is why, in this case, a typical evolutionary process might lead to convergence, freeing up time for further exploration of the space issue. 

### 5.3. Experiment 3: A Self-Assessment with Different Values of ε and τ

The objective of the third set of experiments is to test the TPR, PPV, and F-Score of the model with different values of ε and τ for the PAN 13-14 dataset. As shown in Table 9 and Table 10, the proposed model achieves better results as compared with the GA version in terms of TPR, PPV, and F-Score, which shows a general trend for documents as *θ* and *β* increase, TPR decreases, and PPV increases. At ε=8 and τ=30, the best F-score is obtained for the documents. That means that changing ε and τ will affect the value of the TPR, PPV, and F-Score. The superiority of the *θ* comes from the fact that it helps to minimize false detection. By adjusting the ε parameter, we may reduce the number of document-level comparisons performed during the passing stage and hence the number of plagiarized documents. How much of a sentence from a questionable source is plagiarized is determined by the threshold τ.

### 5.4. Experiment 4: Performance Accuracy with Different Training Samples

The objective of the fourth set of experiments is to test the accuracy of the model with different values of document datasets. As the model has more enrolled samples, the chance of a correct hit increases. The accuracy of the proposed model achieves better results with an increasing number of training documents. For all values of the documents’ number, accuracy increases by approximately 10% on average. This means that if the model is trained with more samples, it will be better at finding plagiarized documents. As shown in Table 11, as expected, the identification rate increases as the number of samples grows. The accuracy rate rises by approximately 10–15% for each increase in the number of samples in the dataset. The accuracy may reach 98% when all samples are used to train the proposed model, owing to the role played by the QGA in determining which characteristics to use. In order to achieve this improvement, the time needed to train the model increases. When compared to the time invested in testing, however, this delay is small. The optimum feature selection module is the most time-consuming part of the training procedure.

### 5.5. Experiment 5: A Comparative Study with Recent Related Work

The fifth set of experiments was also conducted to evaluate the proposed system compared with the recent models. Models from [41,59,60,61,62,63] were selected to compare the proposed model to other well-known methods for text similarity detection. In the study described in [59], plagiarism is only evaluated after two levels of filtering have been applied using the bag-of-words approach, one at the document level and the other at the sentence level. In Ref. [60], a three-stage method based on the Smith–Waterman algorithm for plagiarism detection employs context matching and pre-trained word embeddings to detect instances of synonym substitution and word reordering. By combining linguistic features such as path similarity and depth estimation measures to compute the resemblance between the pair of words and assigning different weights to each feature, the work presented in [61] uses semantic knowledge to detect the plagiarized part of the text.

In Ref. [62], text embedding vectors are used to compare document similarity for plagiarism detection; these vectors include both semantic and syntactic information about the text, and they provide effective text alignment between the suspect and original documents. Sentences with the greatest resemblance are regarded as candidates or seeds of plagiarism cases by comparing their appearances in the source and suspect documents. Syntactic similarities between source and suspect phrases may be revealed using part-of-speech tag n-grams, as shown in [63]. Word2Vec, a word embedding method, is employed to quantify the semantic relatedness between words, while the longest common subsequence approach is used to quantify the semantic similarity between the source and suspect sentences. Table 12 shows the performance results of the proposed system compared to other related systems in terms of precision and F-measure.

The performance results for the PAN 13-14 corpus demonstrate that the proposed system outperforms the state-of-the-art systems on all documents. It can be seen that the majority of the previous systems acquired varying ranks in the various datasets. This variation is due to the structure of the dataset and the kinds of plagiarism that were included in it. However, the suggested method maintained its position as the best across all of the datasets. The suggested approach thus achieves effectiveness and reliability in detecting the various types of textual plagiarism based on these results. They also indicate the ability of the QGA to find the hyperplane equation of the selected features to detect the different types of text similarities. Utilizing the GQA helps to identify the interconnected, cohesive sentences that effectively convey the source document’s main idea with more accuracy. See [64] for a more comparative study of different PD methods. Regarding the running time, we find that there are no major variations between any of the approaches and that the average variance between them is just 4 s. The total time largely depends on the size of the corpus (1535 documents in our case). The suggested approach requires more time, but the results are more precise.

### 5.6. Experiment 6: Run Time and Complexity Analysis 

The last set of experiments is meant to prove that the suggested QGA-based PD model converges quickly compared to the traditional GA-based model for PAN 13–14 datasets with different population sizes. The results shown in Table 13 confirm this fact with an average 1% reduction. As discussed earlier, the total running time largely depends on the size of the corpus.

It is usually true that quantum algorithms may reduce the complexity of their classical counterparts. We can roughly estimate the complexity decrease by comparing the global complexity of the QGA to that of the GA. The global complexity for the QGA is O(N), where *N* is the total population size (Evaluation + Interference). The global complexity of an ordinary GA is often in the order of $O(N^{2})$ (Evaluation + Selection + Crossover + Mutation). Indeed, one can foresee what would occur if we were to study a very large population of chromosomes; the QGA instead of the GA would be extremely beneficial. Our experimental results show that the QGA can be a very promising tool for exploring large search spaces while preserving the relation efficiency/performance. See [22] for more details.

## 6. Conclusions

From the standpoint of a forensic linguist, it is critical to determine with absolute certainty whether a text is an original or the consequence of plagiarism. Expert evidence from a forensic linguist is often required in court cases, but this field is not only concerned with law; forensic linguists also study public-facing topics. Therefore, incorrect judgments must be avoided at all costs to avoid miscarriages of justice, whether in the classroom or the courtroom. In this paper, a new approach based on the semantic similarity concept and the QGA for PD is proposed. The proposed model includes four main steps: the pre-processing and document representation module, sentence-level concept extraction using the QGA, the document-level detection phase, and the passage-level detection phase.

The semantic similarity concept, which depends on intelligent techniques, is employed for extracting the concepts from documents in an effective way to enhance the model’s performance. The QGA is employed to find relatedness between sentences that show the concept of the source document briefly, enhancing the model’s processing time. The solution based on PDS has the advantage of detecting plagiarized ideas in documents presented via summarization.

The proposed model was evaluated by using samples of benchmarked datasets. Based on the obtained results, the proposed model for the detection of plagiarism shows an excellent performance in terms of accuracy. It has been compared with the HGA and the GA-based PD model, and it has come up with better results against them. The QGA has been proven to provide better results in terms of accuracy without adding any complications to the model. The solution’s shortcomings, such as WordNet’s inability to measure all possible semantic relationships between words, reduce its efficiency. Despite the method’s general effectiveness, there are other methods to implement the idea, such as paraphrasing and expanding upon concepts. A possible future study includes making use of a different database to determine how closely related terms are semantically. Furthermore, future work will focus on comparing different QGA strategies to study the effect of choosing rotation gate angles. Another perspective of this work is to study parallel QGAs because QGAs are highly parallelizable.

## Figures and Tables

**Table 1:** Lookup table for quantum gate rotation [22].

$x_{i}$ $b_{i}$	f(x)>f(b)	$\delta \theta_{i}$	$S(a_{i}$ **,** $b_{i})$			
			$a_{i}.b_{i}>0$	$a_{i}.b_{i}<0$	$a_{i}=0$	$b_{i}$ ** = 0**
0 0	0	0.001π	-	+	±	±
0 0	1	0.001π	-	+	±	±
0 1	0	0.08π	-	+	±	±
0 1	1	0.001π	-	+	±	±
1 0	0	0.08π	+	-	±	±
1 0	1	0.001π	+	-	±	±
1 1	0	0.001π	+	-	±	±
1 1	1	0.001π	+	-	±	±

**Table 2:** Data statistics of PAN 13-14.

PAN 13-14 Dataset	Files					
	Training Data		Testing Data-1		Testing Data-2	
	Source	Suspicious	Source	Suspicious	Source	Suspicious
Non-plagiarized (NP)	947	-	97	-	949	-
Plagiarized (P)	238	-	24	-	236	-
Total	1185	237	121	102	1185	237

**Table 3:** Performance metrics [50].

Define	Metric
Accuracy	ACC = $\frac{TP+TN}{TP+TN+FN+FP}=\frac{TP+TN}{P+N}$
Sensitivity or Recall or Hit Rate or True Positive Rate	TPR =$\frac{TP}{TP+FN}=\frac{TP}{P}$
Precision or Positive Predictive Value	PPV $=\;\frac{TP}{TP+FP}$
F-Score	F-Score $=\frac{2*PPV*TPR}{PPV+TPR}=\frac{2TP}{2TP+FP+FN}$

**Table 4:** QGA parameters settings.

Parameter	Value
Population Size (N)	50
Max. No. of Generations (Max_Gen)	10
Selection	Highest Fitness
Probability of Crossover	0.7
Probability of Mutation	0.3
Termination Condition	*Max_Gen*

**Table 5:** Comparison between semantic concept extractions with the QGA, HGA, and GA methods (P: plagiarized, NP: non-plagiarized) for the PAN13-14 dataset. (Average of testing data-1 and testing data-2)

PD Methods	TPR Value		PPV Value		F-Score	
	P	NP	P	NP	P	NP
Semantic concept extractions with the QGA (proposed model)	1	0.99	0.99	0.98	0.99	0.98
Semantic concept extractions with the HGA [58]	0.98	0.97	0.98	0.95	0.98	0.96
Semantic concept extractions with the GA [8]	0.97	0.95	0.96	0.93	0.97	0.94

**Table 6:** Confusion matrix.

		Predicted Class	
		Condition Positive (P)	Condition Negative (N)
Actual Class	Condition Positive (P) The number of real positive cases in the data	True Positive (TP) Correct positive prediction	False Positive (FP) Incorrect positive prediction, Type I error.
	Condition Negative (N) The number of real negative cases in the data	False Negative (FN) Incorrect negative prediction, Type II error	True Negative (TN) Correct negative prediction.

**Table 7:** GA-based PD confusion matrix (average).

		Predicted Class	
		Positive (P)	Negative (N)
Actual Class	Positive (P)	93%	7%
	Negative (N)	7%	93%

**Table 8:** QGA-based PD confusion matrix (average).

		Predicted Class	
		Positive (P)	Negative (N)
Actual Class	Positive (P)	98%	2%
	Negative (N)	2%	98%

**Table 9:** Model performance with different ε values.

*ε* Values	TPR Value		PPV Value		F-Score	
	QGA	GA	QGA	GA	QGA	GA
1	1	0.98	0.23	0.20	0.39	0.38
2	1	0. 98	0.25	0.23	0.41	0.39
3	1	0.98	0.26	0.26	0.44	0.41
4	1	0.98	0.34	0.32	0.53	0.52
5	1	0.97	0.44	0.43	0.62	0.61
6	0.98	0.97	0.73	0.72	0.84	0.81
7	0.98	0.97	0.85	0.84	0.94	0.93
8	0.97	0.97	0.98	0.96	0.97	0.96
9	0.97	0.96	1	0.98	0.95	0.95
10	0.93	0.92	1	1	0.94	0.94

**Table 10:** Model performance with different τ values.

*τ* Values	TPR Value		PPV Value		F-Score	
	QGA	GA	QGA	GA	QGA	GA
10	1	0.97	0.46	0.43	0.64	0.62
15	1	0.97	0.47	0.45	0.66	0.63
20	1	0.97	0.60	0.51	0.74	0.69
25	0.99	0.95	0.62	0.62	0.76	0.75
30	0.95	0.89	0.80	0.82	0.84	0.82
35	0.86	0.65	0.83	0.84	0.77	0.74
40	0.71	0.60	0.85	0.86	0.73	0.71
45	0.58	0.46	0.96	0.96	0.65	0.62
50	0.36	0.27	0.99	0.98	0.44	0.41

**Table 11:** Accuracy of the model with different numbers of samples.

No of Samples	5	10	15	30	50	100	500	750	1000	1535
Accuracy (%)	15	35	40	50	55	60	70	75	80	98

**Table 12:** Comparison results of the proposed text similarity system and other relevant systems in the PAN 13-14 dataset. (Average for testing data-1 and testing data-2.)

PD Methods	Precision (%)	F-Measure (%)	Run Time (Sec)
Arabi, H., Akbari, M [59]	90.08	86.65	56
Alvi, F. et al. [60]	92.52	86.84	55
Ahuja, L. et al. [61]	85.60	88.65	49
Gharavi, E. et al. [62]	89.75	90.15	53
Yalcin, K. et al. [63]	92.76	90.l8	54
El-Rashidy M. et al. [41]	92.61	89.43	51
Proposed System	97.91	94.68	58

**Table 13:** Running time (average) with different population sizes for both the QGA and the traditional GA-based PD model for PAN 13-14.

Population Size	5	10	15	30	50	100
QGA-based PD Model	49	51	52	53	55	58
GA-based PD model	54	56	57	59	60	65

## Data Availability

The datasets for this research are available via https://pan.webis.de/data.html accessed on 1 January 2023.
